# Digital access and maternal healthcare inequalities in Pakistan, 2024–2025: a population-based cross-sectional study

**DOI:** 10.3389/frph.2026.1976275

**Published:** 2026-10-09

**Authors:** Syed Sikandar Shah, Aftab Alam, Junaid Asghar

**Affiliations:** 1 Department of Clinical Pharmacy and Pharmacology, RAK College of Pharmacy, RAK Medical and Health Sciences University, Ras Al Khaimah, United Arab Emirates; 2 Faculty of Pharmacy, Near East University, Nicosia, Türkiye

**Keywords:** maternal healthcare, postnatal care, digital access, socioeconomic inequality, Pakistan

## Abstract

**Background:**

Digital access may influence how women obtain health information, communicate, and navigate healthcare, but it is also strongly shaped by socioeconomic and geographic disadvantage. We examined associations between recent digital access and maternal healthcare utilization among women in Pakistan and assessed whether these associations differed by socioeconomic position.

**Methods:**

We analyzed 2024–25 Household Integrated Economic Survey data from 10,723 ever-married women aged 15–49 years who reported a live birth during the preceding three years in Pakistan's four provinces. Recent digital access during the three months before interview was classified into four mutually exclusive nominal categories: fully disconnected, offline device use, Internet use without any of 11 specified ICT activities, and Internet use with ≥1 recorded ICT activity. Primary outcomes were ≥4 antenatal-care contacts (ANC ≥4), facility delivery, and maternal postnatal care (PNC) within six weeks. Survey-weighted modified Poisson models estimated adjusted prevalence ratios (aPRs), with maternal age modeled using linear and quadratic terms and lifetime live births continuously. Marginal standardization estimated adjusted prevalences and absolute differences.

**Results:**

Fully disconnected women had lower adjusted prevalence of ANC ≥4 than women reporting Internet use with ≥1 recorded ICT activity (aPR 0.75, 95% CI 0.67–0.85), corresponding to standardized prevalences of 47.2% and 58.6%, respectively (difference −11.4 percentage points, 95% CI −13.9 to −8.9). The association with ANC differed across household consumption quintiles (interaction *P* = 0.004). Among women in the poorest quintile, 19.2% reported Internet use with ≥1 recorded ICT activity, compared with 84.2% among women in the richest quintile. Associations were smaller for facility delivery (aPR 0.94, 95% CI 0.89–1.00) and maternal PNC (0.87, 0.76–0.99) and were less consistent across sensitivity analyses. The ANC association remained evident after additional social-context, temporal, missing-data, and finer-geographic analyses.

**Conclusions:**

Complete digital disconnection was consistently associated with lower antenatal-care utilization and was concentrated among socioeconomically disadvantaged women. Associations with facility delivery and maternal PNC were weaker and more context-sensitive, indicating that recent digital access does not relate uniformly to all stages of maternal care. Because digital status was measured near the survey interview and may have changed after the index pregnancy, these findings are associational and do not establish a causal effect of digital access on maternal healthcare use.

## Introduction

Despite substantial progress in maternal survival, pregnancy and childbirth remain important causes of preventable morbidity and mortality worldwide. An estimated 260,000 maternal deaths occurred in 2023, with the burden concentrated disproportionately in low- and middle-income countries (LMICs) (1). Effective antenatal care (ANC), appropriate care during childbirth, and postnatal care (PNC) provide opportunities for prevention, early detection of complications, timely referral, and continuity of care across pregnancy and the postpartum period (2). Yet utilization of these services remains markedly unequal. Recent systematic reviews have identified household economic position, maternal education, and place of residence as persistent determinants of maternal-healthcare utilization in developing countries and South Asia (3, 4). In Pakistan, national analyses likewise demonstrate substantial socioeconomic and geographic inequalities in ANC, skilled or facility-based delivery, PNC, and completion of the maternal-care continuum (5–8).

Rapid digitalization introduces another potentially important dimension to these established inequalities. Digital technologies can influence how people obtain health information, communicate with healthcare providers and family networks, receive reminders, navigate referral or appointment systems, and interact with increasingly digital health services (9–11). However, digital access is not an isolated individual attribute. Connectivity, affordability, device access, literacy, privacy, autonomy, and effective use are themselves socially patterned, and digital systems may reproduce or widen existing inequalities when they are designed around assumptions of continuous connectivity or personal device ownership (9, 10). Accordingly, digital access may operate both as a potential pathway affecting healthcare use and as a marker of broader socioeconomic and geographic advantage.

This distinction is particularly relevant in Pakistan, where national digital infrastructure has expanded rapidly but access remains uneven. By March 2025, Pakistan had approximately 147.2 million broadband subscribers and a broadband penetration rate of 59.8%; cellular mobile services reached about 91% of the population and 3G/4G signals reached more than 81% (12). At the household level, HIES 2024–25 reported Internet availability of approximately 82% in urban areas compared with 61% in rural areas (13). Earlier nationally representative research has also documented pronounced gender, educational, and rural–urban inequalities in personal mobile-phone ownership in Pakistan (14). Similar patterns are observed across LMICs, where women who are poorer or live in rural areas are systematically less likely to own a mobile phone, potentially limiting their ability to benefit from digitally delivered reproductive and maternal-health services (15). Thus, expansion of telecommunications infrastructure does not necessarily imply equal individual access to, control over, or meaningful use of digital resources.

A plausible relationship between digital access and healthcare utilization is supported by several strands of empirical research, although findings arise from different settings and should not be interpreted as directly transferable to Pakistan. Suziedelyte found that Internet health-information seeking complemented rather than substituted for professional healthcare use in the United States (16). In India, Krishnatri and Vellakkal exploited a major telecommunications-market disruption and reported increased utilization of ANC, institutional delivery, PNC, and modern contraception following expansion of affordable Internet access (17). Pregnancy-specific research also shows that women commonly use the Internet to supplement information obtained from healthcare professionals (18). At the same time, digital connectivity is not necessarily beneficial. Health-related Internet use may increase access to information while simultaneously producing information overload (19), and online pregnancy environments can expose women to misinformation or unsupported health claims (20). Digital connectivity should therefore be understood as a potentially dual-edged resource, rather than as an inherently positive exposure.

Evidence from LMIC maternal-health settings further supports the relevance of digital access while emphasizing heterogeneity in both exposure and effect. A systematic review of mobile technologies and maternal and neonatal healthcare found generally favorable associations or intervention effects across several utilization outcomes but also identified variation in study quality, context, phone ownership, socioeconomic access, and technology use (21). In Pakistan, Feroz et al. demonstrated that access to and willingness to use mobile-phone voice messaging for ANC differed among women in rural Sindh, illustrating both the potential reach of low-bandwidth digital approaches and the importance of understanding who can actually access them (22). These studies are informative but address health-specific mobile technologies or interventions; they are not equivalent to the broader patterns of device and Internet access measured in population surveys.

Conceptually, the present study therefore treats digital access as situated within a wider system of determinants rather than as an independent technological “treatment.” Socioeconomic position, education, employment, household resources, residence, women's social circumstances, telecommunications infrastructure, and local health-system conditions may shape both digital access and maternal healthcare utilization. Conditional on these shared determinants, digital access could plausibly relate to care through information acquisition, communication, social support, appointment or referral navigation, and other service-access functions. Conversely, misinformation, information overload, lack of privacy, mediated or shared access, or substitution of online advice for professional consultation could operate in the opposite direction (9–11, 16, 18–20). These pathways are hypothesized mechanisms derived from prior literature; the cross-sectional data used in this study do not identify them as causal mediators.

Against this background, an important empirical question remains unresolved in Pakistan. Previous national maternal-health research has primarily examined socioeconomic, demographic, autonomy-related, and geographic determinants of service utilization (5–8), while research on digital access has focused largely on phone ownership, the gender digital divide, or specific mHealth applications (14, 22). It is therefore unclear whether women with limited recent digital access also have lower maternal-healthcare utilization after accounting for major conventional socioeconomic and geographic determinants, whether any such association differs across stages of maternal care, and whether it is stronger or weaker across socioeconomic groups.

We addressed these questions among women with a recent live birth in Pakistan's four provinces. Recent digital access was represented by four nominal patterns of observed device, Internet, and specified ICT use, rather than by an assumed hierarchy of digital competence. We prespecified ANC ≥4, facility delivery, and maternal PNC within six weeks as the three primary utilization outcomes and evaluated socioeconomic effect modification using household consumption quintile. We hypothesized that complete digital disconnection would identify women with lower maternal-healthcare utilization after adjustment for established demographic, socioeconomic, household, residential, and geographic factors, while recognizing that associations could differ across care stages and socioeconomic groups. Because the ICT measures refer to the three months preceding interview and may therefore postdate the index pregnancy, the study was explicitly designed to estimate associations rather than causal effects and to test the robustness of those associations to social-context, temporal, missing-data, and finer-geographic specifications.

## Materials and methods

### Study design and data source

We conducted a population-based cross-sectional analysis of the Pakistan Household Integrated Economic Survey (HIES) 2024–25, implemented by the Pakistan Bureau of Statistics (PBS). HIES collects individual- and household-level information on demographic characteristics, education, employment, information and communication technology (ICT), health and maternity care, housing, assets, consumption expenditure, and other socioeconomic domains (13, 23).

The present analysis used the four-province sample covering Punjab, Sindh, Khyber Pakhtunkhwa, and Balochistan; Islamabad Capital Territory was incorporated within Punjab in accordance with PBS reporting conventions. HIES used a stratified two-stage sampling design based on the updated 2023 Population and Housing Census sampling frame. In the first stage, urban enumeration blocks and rural village blocks were selected as primary sampling units (PSUs) using probability proportional to size. Households were then selected systematically from updated household listings. The four-province sample comprised 30,123 households in 2,153 PSUs distributed across 158 survey strata (13, 23).

### Study population

The HIES maternity module was administered to ever-married women aged 15–49 years. For the present study, eligible participants were women who were present for interview and reported a live birth during the preceding three years. Maternal-care questions referred to the most recent relevant birth.

Individual records were linked across the household roster, ICT, education, employment, maternity, decision-making, household-assets, and consumption files using PBS household and individual identifiers. Data auditing confirmed successful linkage to the individual ICT data for all women meeting the maternal-health eligibility criteria.

The final primary cohort comprised 10,723 women. All eligible women were retained; no separate sample-size calculation was performed because the analysis used the complete eligible HIES sample.

### Recent digital-access measure

The primary exposure was recent digital access and use during the three months preceding interview. The HIES ICT module separately records mobile/smartphone use and ownership, desktop/laptop/tablet use, Internet use, and performance of 11 specified ICT activities (23).

Women were classified into four mutually exclusive categories:

1. Fully disconnected: no reported mobile/smartphone use, desktop/laptop/tablet use, or Internet use during the preceding three months.

2. Offline device use: mobile/smartphone and/or computer-device use but no Internet use.

3. Internet use without recorded ICT activity: Internet use but none of the 11 specified ICT activities recorded.

4. Internet use with ≥1 recorded ICT activity: Internet use together with at least one of the 11 specified ICT activities.

The fourth category served as the reference group in regression analyses.

These categories were treated as nominal patterns of recent digital access and use, not as an ordinal scale or a validated measure of digital literacy, competence, engagement, or skill. The 11 HIES activities capture selected ICT behaviors; Internet users may undertake other online activities not represented by these items. Consequently, Internet use without a recorded ICT activity was not interpreted as lower digital competence, and reporting one or more recorded activities was not interpreted as evidence of advanced digital literacy.

The primary classification was based principally on reported technology use rather than personal ownership. Internet access could therefore occur through a personally owned device, another household member's device, a shared device, or another Internet-enabled platform. Personal phone ownership, smartphone ownership, and Internet use were examined separately in sensitivity analyses (Supplementary Table S1).

Online health-information seeking and online healthcare appointments were not incorporated into the exposure definition because these behaviors may arise in response to health needs and their temporal relationship with the earlier maternity episode cannot be established. They were therefore examined descriptively rather than interpreted as mediators of the primary associations.

### Maternal healthcare outcomes

Three outcomes representing different stages of maternal healthcare utilization were defined as the primary outcomes: ANC ≥4, facility delivery, and maternal PNC within six weeks.

### Antenatal care

HIES records whether prenatal consultation was received and the reported number of consultations. The primary ANC outcome was receipt of at least four ANC contacts (ANC ≥4). Women reporting no prenatal consultation were classified as not attaining this outcome.

ANC ≥4 was retained as the primary utilization threshold because it permits stable estimation and comparison with the extensive maternal-health literature based on the historical four-contact framework. It was not interpreted as the current WHO recommendation for adequate ANC. In accordance with current WHO guidance, ANC ≥8 was examined as an important secondary outcome (2). Any ANC and first-trimester initiation were also considered secondary ANC indicators.

### Facility delivery

Facility delivery was defined as childbirth in a government hospital or clinic or a private hospital or clinic. Births occurring at home or at locations classified as “other” were categorized as non-facility deliveries.

This outcome measures place of delivery and healthcare utilization only. It does not measure technical quality of care, clinical appropriateness, respectful maternity care, emergency obstetric readiness, or maternal or neonatal outcomes.

### Maternal postnatal care

Maternal PNC was defined as receipt by the woman of a postnatal check-up within six weeks of delivery, either at a healthcare facility or at home, consistent with the wording of the HIES maternity questionnaire (23).

The released HIES maternity module does not contain a separately identifiable newborn PNC variable suitable for parallel analysis. Newborn PNC was therefore not inferred from breastfeeding, delivery, or other child-related variables.

### Sequential maternal care continuum

Continuity of maternal healthcare was examined using a sequential cumulative framework rather than relying only on a single all-or-none composite outcome. Three successive stages were evaluated:

ANC ≥4 → ANC ≥4 plus facility delivery → ANC ≥4 plus facility delivery plus maternal PNC.

Survey-weighted conditional transition proportions were additionally estimated for facility delivery among women who had achieved ANC ≥4 and for PNC among women who had achieved both ANC ≥4 and facility delivery.

Adjusted marginal prevalences of each cumulative stage were estimated across the four digital-access categories and displayed as a maternal-care cascade. A binary indicator representing completion of all three components was retained as a secondary summary measure.

### Socioeconomic position and covariates

Household socioeconomic position was measured using monthly per-capita consumption expenditure. Consumption components were harmonized across their HIES reporting periods following PBS procedures, and households were classified according to the official HIES 2024–25 national consumption-quintile thresholds, from the poorest to the richest quintile (13).

Covariates were selected *a priori* on substantive grounds, informed by the conceptual framework and established determinants of maternal healthcare and digital access, rather than according to univariable statistical significance.

The fully adjusted primary model included:

- maternal age, modeled continuously using linear and quadratic terms;
- lifetime number of live births, modeled continuously;
- current marital status;
- educational attainment;
- employment status;
- household consumption quintile;
- household size;
- rural/urban residence; and
- province fixed effects.

Maternal age was represented by age and age^2^ to allow a nonlinear relationship with healthcare utilization without imposing arbitrary categorical boundaries. Lifetime live births entered the primary model as a continuous integer. Categorical specifications of maternal age and lifetime live births were evaluated in functional-form sensitivity analyses.

Province was represented by indicator variables and modeled as province fixed effects.

### Social-context sensitivity analyses

Women's household decision-making and traditional media resources may be associated with both digital access and maternal healthcare utilization. Sensitivity analyses therefore examined the stability of the primary estimates after additional adjustment for:

- women's participation in decisions concerning household medical treatment expenditure;
- women's participation in decisions concerning paid employment; and
- household radio/television availability.

These variables were evaluated individually and jointly. They were not incorporated automatically into the primary model because they were measured at interviews and could themselves have changed after a pregnancy occurring up to three years earlier. Their inclusion therefore assessed sensitivity to additional measured social context rather than implying complete or temporally ordered control of confounding.

### Geographic and health-system context

Province fixed effects and rural/urban residence were included in the primary model to account for broad geographic differences.

The released HIES data do not directly measure maternity-facility readiness, local provider density, travel time to services, or consistently identifiable exposure to district-level health-financing and service-delivery programs. In addition, the HIES processing code does not provide a uniform national district identifier: depending on the geographic stratum, the coding structure may correspond to an administrative division or district. Reliable nationwide linkage to external district-level policy or health-system datasets was therefore not possible without introducing geographic misclassification.

To evaluate sensitivity to finer local context, the province fixed effects in the primary model were replaced by fixed effects for the 158 HIES sampling strata. These fixed effects provide substantially finer geographic adjustment but were not interpreted as direct measures of provider supply, facility readiness, transport accessibility, or health-policy exposure.

### Statistical analysis

All analyses incorporated the HIES complex survey design. PBS sampling weights were applied without rescaling, and the maternal cohort was analyzed as a survey subpopulation so that the underlying design structure was preserved for variance estimation.

Variance estimation accounted for:

- survey weights;
- 158 sampling strata; and
- clustering within 2,153 PSUs.

Nine singleton strata were handled using a centered singleton-stratum approach. The resulting survey design contained 1,995 design degrees of freedom.

Survey-weighted percentages and 95% confidence intervals were estimated for digital-access categories, maternal-health outcomes, and participant characteristics. Unweighted sample counts were reported separately from survey-weighted percentages.

Because the primary binary outcomes were common, associations were estimated using survey-weighted modified Poisson regression with a log link and design-based robust sandwich variance, allowing direct estimation of prevalence ratios (24). For woman *i*, the fully adjusted model can be expressed as:

$$\log\{E(Y_{i},|,D_{i}X_{i}P_{i})\}=\beta_{0}+\overset{3}{\underset{k=1}{\sum }}\beta_{k}I(D_{i}=k)+\beta_{4}Age_{i}+\beta_{5}Age_{i}^{2}+\beta_{6}Births_{i}+\gamma^{T}X_{i}+\delta^{T}P_{i}$$

where $Y_{i}$ represents the binary maternal-health outcome; $D_{i}$ represents the four-category recent digital-access measure, with Internet use plus ≥1 recorded ICT activity as the reference; $Age_{i}$ and $Age_{i}^{2}$ represent maternal age and its quadratic term; $Births_{i}$ represents lifetime live births; $X_{i}$ contains marital status, education, employment, household consumption quintile, household size, and rural/urban residence; and $P_{i}$ represents province fixed effects.

Because the model used a generalized linear model with a log link, no additive homoskedastic error term was assumed. Statistical uncertainty was estimated from the survey-design robust covariance matrix incorporating weights, strata, and PSU clustering. Standard errors were therefore not clustered at the province level.

Results are reported as prevalence ratios or adjusted prevalence ratios (aPRs) with 95% confidence intervals.

### Marginal standardization

Relative measures were complemented by adjusted prevalence estimates and absolute prevalence differences.

Because predicted values from log-link Poisson models are not inherently constrained to the probability range of 0–1, marginal prevalences were estimated separately using survey-weighted logistic regression with the same adjustment set as the corresponding primary modified Poisson model.

For marginal standardization, all women in the analytical population were assigned in turn to each digital-access category while retaining their observed values for all other covariates. Predicted probabilities were then averaged over the survey-weighted analytical population. Differences between these standardized prevalences provided adjusted absolute prevalence differences.

Confidence intervals for standardized estimates and contrasts were derived from the design-based covariance matrix using the delta method.

### Socioeconomic effect modification

The principal prespecified effect-modification analysis examined whether the association between recent digital access and maternal healthcare utilization differed across household consumption quintiles.

Digital-access category  ×  consumption-quintile interaction terms were added to the fully adjusted modified Poisson model and evaluated using a joint Wald test. Corresponding survey-weighted logistic interaction models were marginally standardized to estimate adjusted prevalences for selected joint combinations of digital access and socioeconomic position.

Interaction results were interpreted as heterogeneity in an observational association, not as evidence that digital intervention would produce different causal effects across socioeconomic groups.

Digital-access  ×  province and digital-access  ×  rural/urban residence interactions were examined as secondary exploratory analyses. Province-specific estimates were reported to aid interpretation, sparse strata were interpreted cautiously, and no multiplicity adjustment was applied to these secondary interaction tests.

### Temporal alignment and birth-date sensitivity analyses

Temporal alignment was a central design limitation because the ICT variables primarily describe the three months preceding interview, whereas maternal-care outcomes may relate to a birth occurring at any time during the preceding three years. Current digital access therefore cannot be assumed to represent digital circumstances during the index pregnancy.

Birth-history information was audited before temporal sensitivity analyses. Restrictions to births occurring within ≤12 or ≤24 months could not be applied reliably across the full cohort because a substantial proportion of records contained unavailable or internally inconsistent birth-month information. Importantly, completeness of birth-date information differed across digital-access categories, so restricting the primary cohort to complete month-level dates could itself introduce differential selection.

Two complementary temporal sensitivity analyses were therefore conducted. First, among women for whom a defensible childbirth year could be established, models additionally incorporated child-birth-year fixed effects to account for aggregate differences across birth cohorts. Second, analyses were repeated among the more restricted subset with internally consistent month and year information placing the birth within the documented three-year eligibility interval.

The internally consistent date subset was used as a sensitivity analysis rather than replacing the primary cohort. Month-by-year fixed effects were not imposed because birth-month information was insufficiently complete and internally consistent for reliable full-cohort estimation.

The direction of bias produced by the exposure–outcome timing mismatch cannot be specified *a priori*. Contemporary digital status may imperfectly classify pregnancy-period digital circumstances, whereas postpartum changes in employment, household resources, phone ownership, or Internet access may be related to prior healthcare utilization. The resulting bias could therefore operate in either direction.

### Missing data

Questionnaire skip patterns were distinguished from genuinely missing observations during data preparation. The recent digital-access measure and principal survey-design variables were complete.

Primary regression models used outcome-specific complete-case analysis, with the number of women contributing to each model reported explicitly in the Results.

Because covariate missingness was limited but non-zero, two additional analyses were undertaken:

1. An inverse-probability-of-complete-case weighting sensitivity analysis; and
2. A complementary analysis retaining explicit missing categories where methodologically appropriate.

These analyses were used to assess whether restriction to complete cases materially altered the primary estimates and were not regarded as eliminating bias from unmeasured causes of missingness.

### Additional sensitivity and secondary analyses

Sensitivity analyses were selected to evaluate specific assumptions concerning exposure measurement, functional form, social context, temporal alignment, geography, socioeconomic classification, and missing data.

They included:

- personal Internet use as an alternative digital exposure;
- mobile/smartphone ownership and personal phone ownership as alternative exposure measures;
- categorical rather than continuous specifications of maternal age and lifetime live births;
- additional adjustment for women's decision-making and household media availability;
- child-birth-year fixed effects;
- restriction to internally consistent birth dates;
- 158 sampling-stratum fixed effects;
- alternative consumption-quintile construction;
- inverse-probability complete-case weighting and missing-category analyses;
- ANC ≥8 as a secondary ANC outcome; and
- Secondary maternal-care indicators, including any ANC, early ANC, skilled birth attendance, and completion of all three primary care components.

These analyses were undertaken to assess the robustness of substantive conclusions to identifiable methodological assumptions, rather than to search across model specifications for statistically significant findings.

### Software and reporting

Data management and statistical analyses were conducted using **Python 3.13.5**, including pandas 2.2.3, NumPy 2.3.5, SciPy 1.17.0, and statsmodels 0.14.6. Figures were generated using Matplotlib 3.10.8.

The primary exposure, three primary maternal-health outcomes, principal covariate structure, and socioeconomic interaction were defined as the central analytical components of the study; secondary and sensitivity analyses were used to evaluate specific assumptions concerning measurement and model specification.

Reporting followed the Strengthening the Reporting of Observational Studies in Epidemiology (STROBE) recommendations for cross-sectional studies (25).

### Ethical considerations

HIES 2024–25 was conducted by the Pakistan Bureau of Statistics. The official survey manual requires informed consent before initiation of the household interview (23). The present study involved secondary analysis of de-identified respondent-level microdata and no direct participant contact.

The secondary analysis was reviewed by the Ethical Review Board of Gomal University and received ethical approval/waiver under Ref. No. 747-21/ERB/GU.

## Results

The HIES 2024–25 household roster included 44,154 women aged 15–49 years. Of 29,601 ever-married women represented in the maternity module, 27,739 were present and interviewed. Among these women, 10,723 reported a live birth during the preceding three years and met the eligibility criteria for the present analysis. All 10,723 women were successfully linked to the individual ICT data and constituted the primary analytical cohort. The derivation of the study population is summarized in Figure 1.

**Figure 1:**
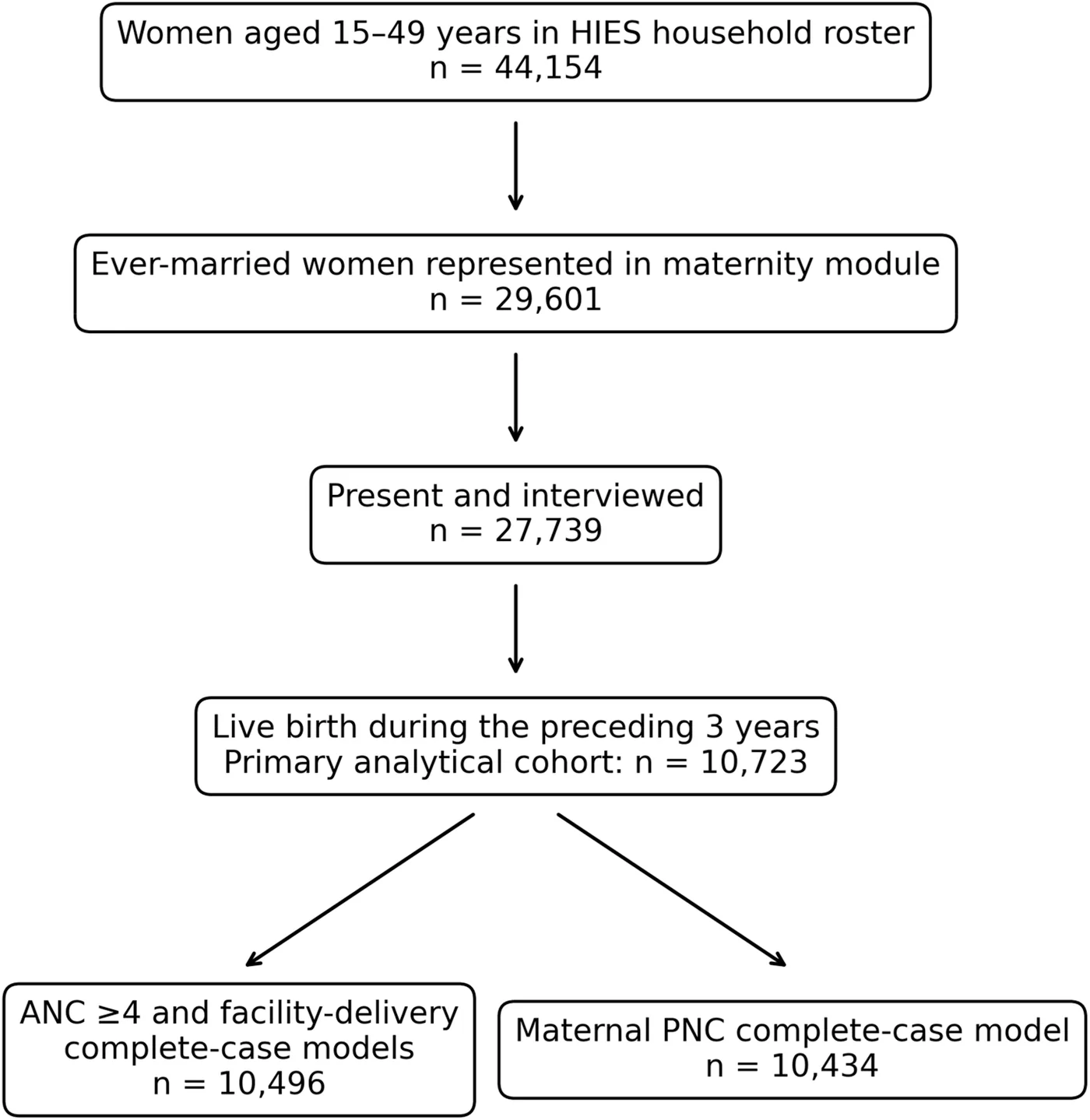
Analytical sample flow. ANC = antenatal care; HIES = Household Integrated Economic Survey; ICT = information and communication technology; PNC = postnatal care. Confidence intervals are not applicable because the figure displays observed sample counts rather than estimated prevalences.

The recent digital-access measure was complete for all women. Lifetime number of live births was missing for 185 women (1.7%), education for 42 (0.4%), and maternal PNC for 63 (0.6%). Accordingly, 10,496 women contributed to the ANC ≥4 and facility-delivery models, and 10,434 contributed to the PNC model (Supplementary Table S7). Selected sociodemographic and household characteristics of the analytical cohort, overall and according to recent digital-access category, are presented in Table 1.

**Table 1:** Selected characteristics of the analytical cohort, overall and by recent digital-access category.

Characteristic	Overall *n* = 10,723	Fully disconnected *n* = 876	Offline device use *n* = 4,070	Internet use, no recorded ICT activity *n* = 1,123	Internet use + ≥ 1 recorded ICT activity *n* = 4,654
Age 15–19 years	2.9	3.8	2.9	3.8	2.5
Age 40–49 years	8.3	11.1	11.0	7.8	6.0
≥4 lifetime live births	34.2	41.4	44.0	35.3	25.8
No formal schooling	48.7	79.0	69.7	58.6	27.0
Higher education (grade ≥11)	16.2	2.8	4.5	7.3	28.5
Employed	18.0	19.3	26.3	16.6	12.2
Q1 poorest consumption quintile	24.0	42.6	39.3	27.7	9.6
Q5 richest consumption quintile	14.5	4.5	4.4	4.5	25.4
Rural residence	64.4	78.9	78.5	67.9	51.5

Values are survey-weighted percentages; unweighted sample sizes are shown in column headings. Digital-access categories are mutually exclusive and refer to the three months preceding interview.

### Recent digital access and maternal healthcare utilization

Among the 10,723 women, 876 were fully disconnected, 4,070 reported offline device use, 1,123 reported Internet use without any of the 11 specified ICT activities, and 4,654 reported Internet use with ≥1 specified ICT activity. Their survey-weighted proportions were 6.8%, 34.8%, 10.3%, and 48.1%, respectively. Digital access was strongly socially patterned: within the poorest consumption quintile, 19.2% of women reported Internet use with ≥1 recorded ICT activity compared with 84.2% in the richest quintile, whereas complete disconnection declined from 12.0% to 2.1%. Complete disconnection was also more frequent in rural than urban areas (8.3% vs. 4.0%) and was highest in Balochistan (18.8%) (Figure 2).

**Figure 2:**
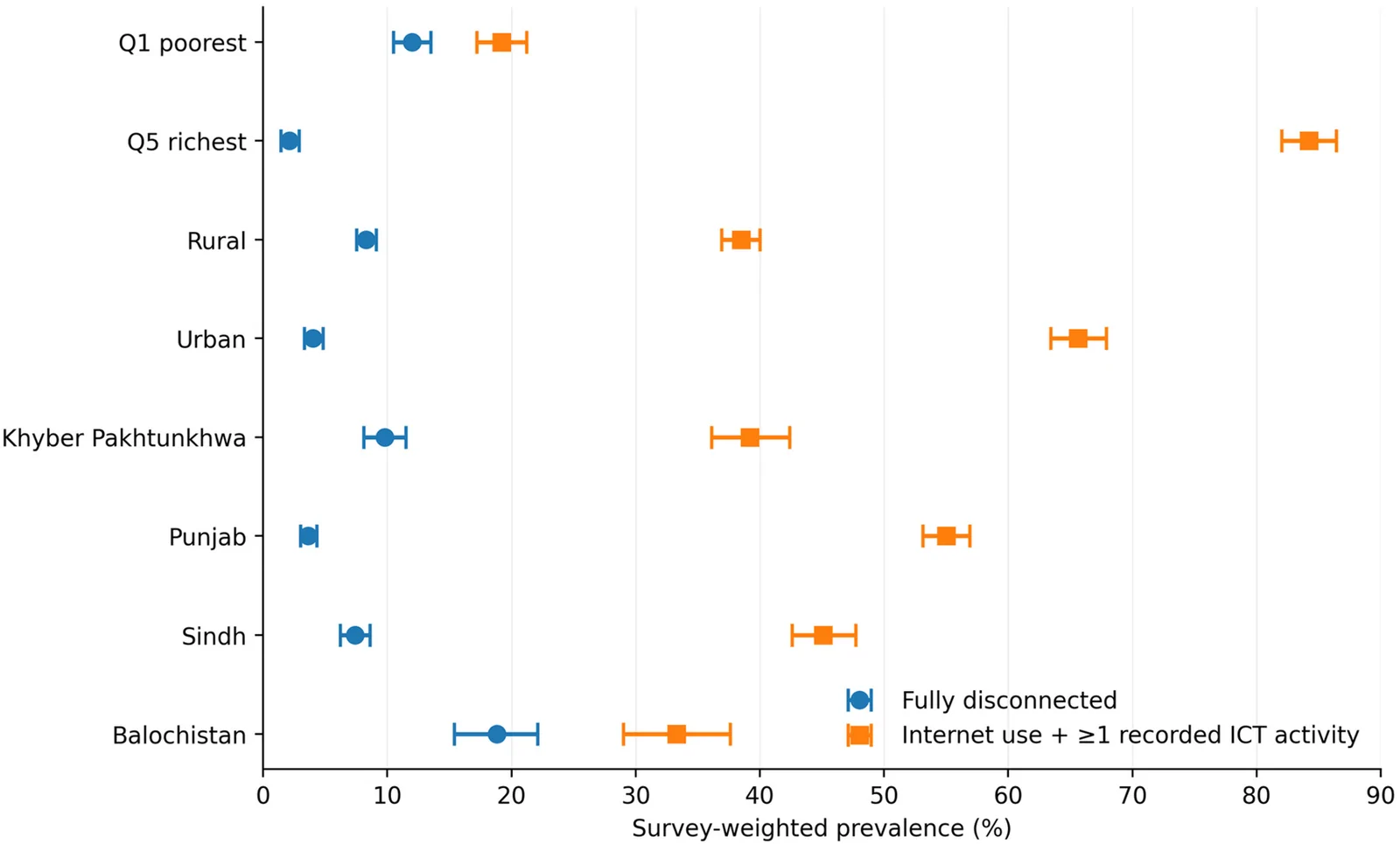
Social and geographic patterning of recent digital access. Points show survey-weighted prevalences of complete digital disconnection and Internet use with ≥1 recorded ICT activity within each subgroup; horizontal error bars show 95% confidence intervals.

Internet use did not necessarily imply personal device ownership. Among 5,777 Internet users, 3,121 did not personally own a phone (survey-weighted 50.7%, 95% CI 49.0–52.3). The corresponding prevalence was 93.7% (91.9–95.3) among Internet users without a recorded ICT activity and 41.5% (39.7–43.3) among Internet users with ≥1 recorded ICT activity. These data identify a distinction between Internet use and personal ownership but do not reveal the exact source of access.

Overall survey-weighted prevalence was 55.2% for ANC ≥4, 83.4% for facility delivery, and 43.5% for maternal PNC within six weeks. Before adjustment, ANC ≥4 prevalence across the four digital-access categories was 31.9%, 43.5%, 52.1%, and 67.6%, respectively. Corresponding prevalences were 71.4%, 77.0%, 81.5%, and 90.2% for facility delivery and 29.7%, 37.2%, 39.3%, and 51.0% for PNC (Supplementary Table S6).

### Primary adjusted associations

In the primary model, which included maternal age and age^2^ and modeled lifetime live births continuously, the clearest association was observed for ANC ≥4. Compared with women reporting Internet use with ≥1 recorded ICT activity, fully disconnected women had lower adjusted prevalence of ANC ≥4 (aPR 0.75, 95% CI 0.67–0.85), as did women with offline device use (0.89, 0.85–0.94). Internet use without any of the specified ICT activities was closer to the reference category (0.96, 0.89–1.03). Marginal standardization estimated ANC ≥4 prevalence of 47.2% among fully disconnected women and 58.6% in the reference category, corresponding to an adjusted difference of −11.4 percentage points (95% CI −13.9 to −8.9) (Table 2; Figure 3).

**Table 2:** Adjusted associations between recent digital access and primary maternal healthcare outcomes.

Outcome	Digital-access category	aPR (95% CI)	Standardized prevalence, % (95% CI)	Difference vs. reference, pp (95% CI)
ANC ≥4	Fully disconnected	0.75 (0.67–0.85)	47.2 (44.7–49.6)	−11.4 (−13.9 to −8.9)
ANC ≥4	Offline device use	0.89 (0.85–0.94)	52.6 (51.8–53.4)	−6.0 (−7.0 to −5.0)
ANC ≥4	Internet use, no recorded ICT activity	0.96 (0.89–1.03)	55.3 (53.9–56.7)	−3.3 (−4.7 to −1.8)
ANC ≥4	Internet use + ≥ 1 recorded ICT activity	1.00 (Ref)	58.6 (57.8–59.3)	Reference
Facility delivery	Fully disconnected	0.94 (0.89–1.00)	81.2 (80.5–81.9)	−3.8 (−4.5 to −3.1)
Facility delivery	Offline device use	0.97 (0.95–0.99)	82.4 (82.1–82.7)	−2.6 (−2.9 to −2.3)
Facility delivery	Internet use, no recorded ICT activity	0.99 (0.95–1.02)	83.1 (82.7–83.6)	−1.9 (−2.3 to −1.5)
Facility delivery	Internet use + ≥ 1 recorded ICT activity	1.00 (Ref)	85.0 (84.8–85.3)	Reference
PNC within 6 weeks	Fully disconnected	0.87 (0.76–0.99)	39.1 (36.5–41.6)	−4.7 (−7.5 to −1.9)
PNC within 6 weeks	Offline device use	1.00 (0.94–1.07)	43.8 (42.6–44.9)	0.0 (−1.5 to 1.5)
PNC within 6 weeks	Internet use, no recorded ICT activity	1.01 (0.92–1.11)	44.2 (42.3–46.0)	0.4 (−1.7 to 2.5)
PNC within 6 weeks	Internet use + ≥ 1 recorded ICT activity	1.00 (Ref)	43.8 (42.8–44.7)	Reference

aPR, adjusted prevalence ratio; p*p*, percentage points; PNC, postnatal care. The modified Poisson model included maternal age and age^2^, lifetime live births continuously, marital status, education, employment, consumption quintile, household size, rural/urban residence, and province fixed effects. Internet use with ≥1 recorded ICT activity was the reference. Standardized prevalences are marginal predictions from survey-weighted logistic models with the corresponding adjustment set.

**Figure 3:**
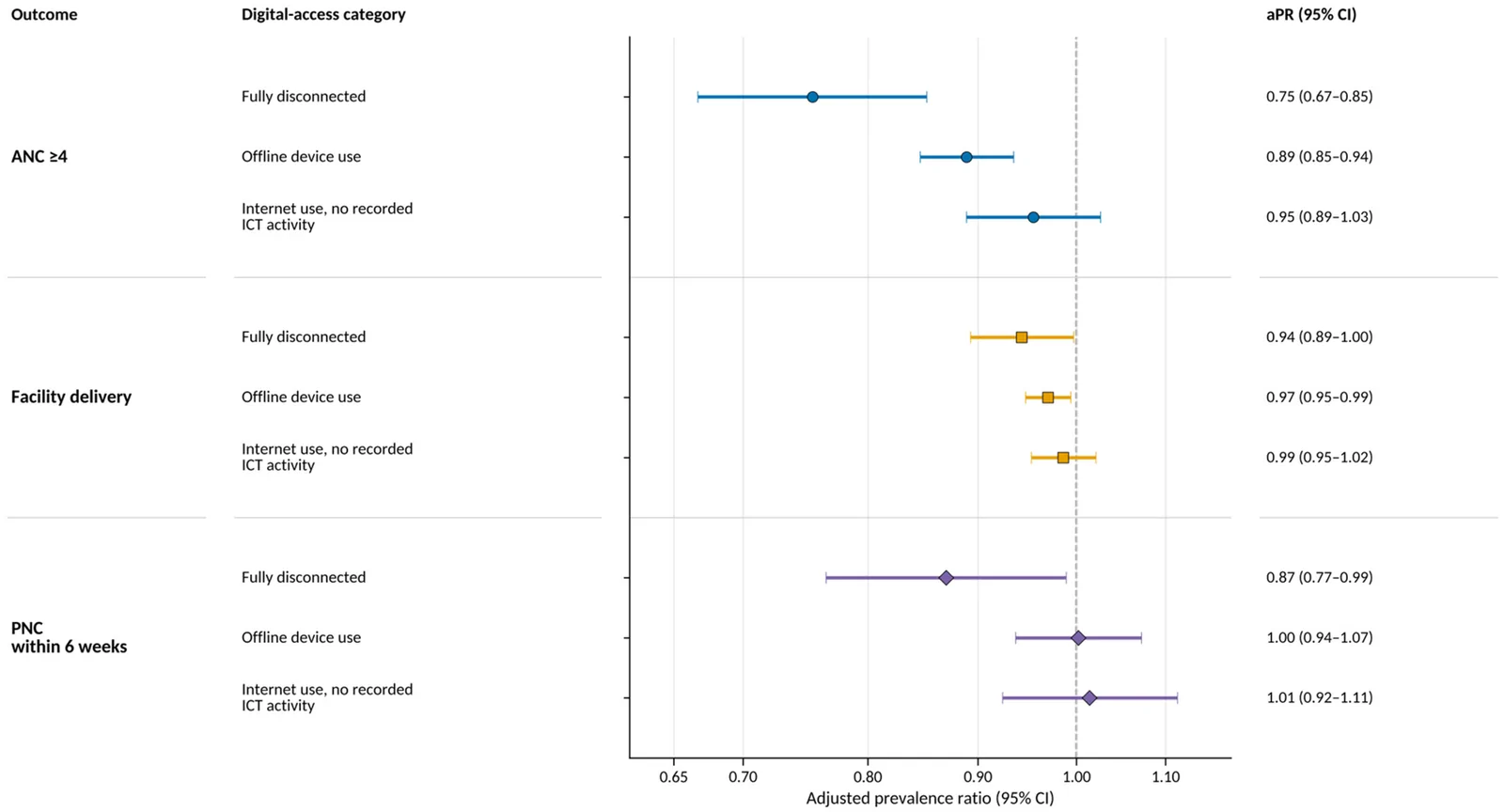
Adjusted associations between recent digital access and the three primary maternal healthcare outcomes. Points show adjusted prevalence ratios (aPRs); horizontal error bars show 95% confidence intervals. The reference category was Internet use with ≥1 recorded ICT activity. The dashed vertical line marks the null value (aPR = 1.00).

Associations with the other primary outcomes were smaller. For facility delivery, the aPRs were 0.94 (95% CI 0.89–1.00), 0.97 (0.95–0.99), and 0.99 (0.95–1.02) for fully disconnected women, offline device use, and Internet use without recorded ICT activity, respectively. For PNC, the corresponding estimates were 0.87 (0.76–0.99), 1.00 (0.94–1.07), and 1.01 (0.92–1.11). Thus, adjusted separation across digital-access categories was largest and most consistent for ANC ≥4, while the facility-delivery association was small and the PNC pattern was largely confined to complete disconnection.

### Socioeconomic inequality and effect modification

Maternal healthcare remained socioeconomically patterned after adjustment for digital access and other covariates. Adjusted ANC ≥4 prevalence was 46.7% in the poorest and 63.5% in the richest consumption quintile, an absolute difference of 16.8 percentage points; corresponding adjusted differences were 12.1 percentage points for facility delivery and 16.5 percentage points for PNC.

The prespecified digital-access  ×  consumption-quintile interaction was supported for ANC ≥4 (joint Wald *P* = 0.004), but not for facility delivery (*P* = 0.216) or PNC (*P* = 0.527). Among women in the poorest quintile, standardized ANC ≥4 prevalence was 36.7% (95% CI 32.5–41.0) among fully disconnected women and 53.4% (51.1–55.7) among women reporting Internet use with ≥1 recorded ICT activity. Expressed as fully disconnected minus the reference category, the adjusted difference was −16.7 percentage points (95% CI −21.5 to −11.9). In the richest quintile, the corresponding standardized prevalences were 56.2% (48.7–63.7) and 66.6% (65.6–67.6), a difference of −10.4 percentage points (−18.0 to −2.9). These estimates describe heterogeneity in association and do not estimate differential causal effects of a digital intervention (Table 3; Figure 4; Supplementary Table S3).

**Table 3:** Socioeconomic inequality, effect modification, and selected joint ANC ≥4 estimates.

Outcome/joint group	Adjusted prevalence, % (95% CI)	Adjusted contrast/interaction
ANC ≥4: Q1 poorest	46.7 (45.5–48.0)	Q5–Q1: +16.8 pp (15.1–18.4); Q5 vs. Q1 aPR 1.40 (1.29–1.52)
Facility delivery: Q1 poorest	78.3 (77.8–78.8)	Q5–Q1: +12.1 pp (11.6–12.6); Q5 vs. Q1 aPR 1.16 (1.12–1.21)
PNC: Q1 poorest	38.5 (36.9–40.0)	Q5–Q1: +16.5 pp (14.2–18.8); Q5 vs. Q1 aPR 1.40 (1.26–1.55)
Digital access × consumption quintile: ANC ≥4	—	Joint Wald *P* = 0.004
Digital access × consumption quintile: facility delivery	—	Joint Wald *P* = 0.216
Digital access × consumption quintile: PNC	—	Joint Wald *P* = 0.527
Q1 poorest + fully disconnected	36.7 (32.5–41.0)	—
Q1 poorest + Internet use + ≥ 1 ICT activity	53.4 (51.1–55.7)	Fully disconnected−reference: −16.7 pp (−21.5 to −11.9)
Q5 richest + fully disconnected	56.2 (48.7–63.7)	—
Q5 richest + Internet use + ≥ 1 ICT activity	66.6 (65.6–67.6)	Fully disconnected−reference: −10.4 pp (−18.0 to −2.9)

Q1, poorest consumption quintile; Q5, richest quintile; pp = percentage points. Interaction *P* values are joint Wald tests. Joint standardized estimates are from the corresponding interaction model.

**Figure 4:**
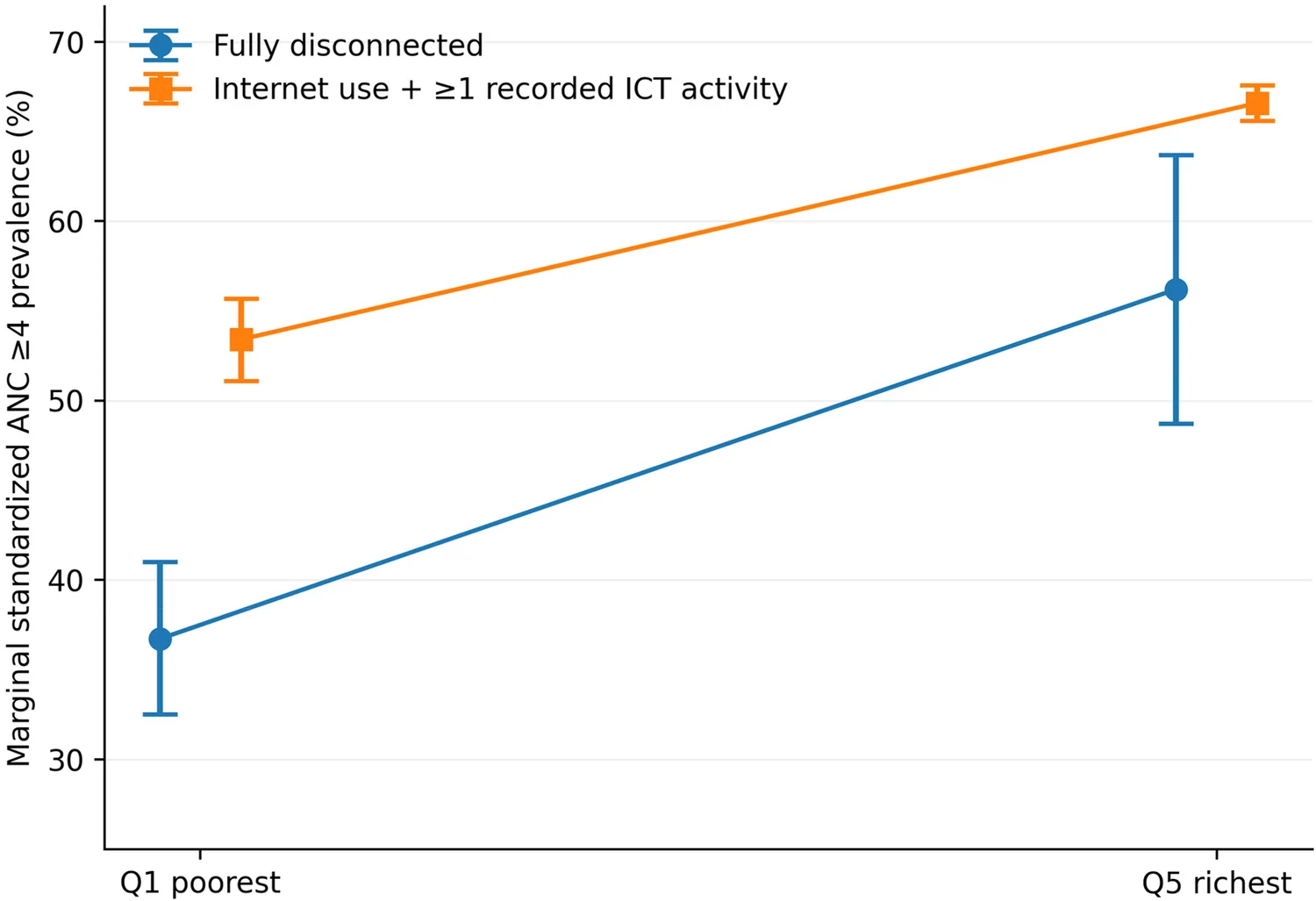
Socioeconomic variation in the association between recent digital access and ANC ≥4. Points show marginal standardized prevalences for selected digital-access categories in the poorest (Q1) and richest (Q5) consumption quintiles; vertical error bars show 95% confidence intervals. The formal interaction test included all four digital-access categories and all five consumption quintiles (joint Wald *P* = 0.004).

### Sequential maternal-care continuum

Sequential analysis localized where differences emerged along the maternal-care pathway. Adjusted standardized prevalence of ANC ≥4 was 47.2%, 52.6%, 55.3%, and 58.6% across the four digital-access categories. After additionally requiring facility delivery, corresponding cumulative prevalences were 43.3%, 47.7%, 51.8%, and 54.2%. Completion of all three components—ANC ≥4, facility delivery, and maternal PNC—was 20.2%, 26.4%, 28.8%, and 29.5%, respectively (Table 4; Figure 5).

**Table 4:** Adjusted maternal-care progression and conditional transition proportions by recent digital-access category.

Digital-access category	ANC ≥4, % (95% CI)	ANC ≥4 + facility, % (95% CI)	ANC ≥4 + facility + PNC, % (95% CI)	Facility | ANC ≥4, %	PNC | ANC ≥4 + facility, %
Fully disconnected	47.2 (42.9–51.4)	43.3 (39.0–47.7)	20.2 (16.1–24.2)	89.5	39.8
Offline device use	52.6 (50.8–54.4)	47.7 (45.9–49.5)	26.4 (24.5–28.3)	88.1	50.4
Internet use, no recorded ICT activity	55.3 (52.0–58.6)	51.8 (48.5–55.1)	28.8 (25.8–31.9)	92.2	50.1
Internet use + ≥ 1 recorded ICT activity	58.6 (56.8–60.4)	54.2 (52.4–55.9)	29.5 (27.9–31.0)	94.4	58.7

The first three columns are marginal standardized cumulative prevalences from adjusted cascade models. The final two columns are unadjusted survey-weighted conditional transition proportions.

ANC, antenatal care; PNC, postnatal care.

**Figure 5:**
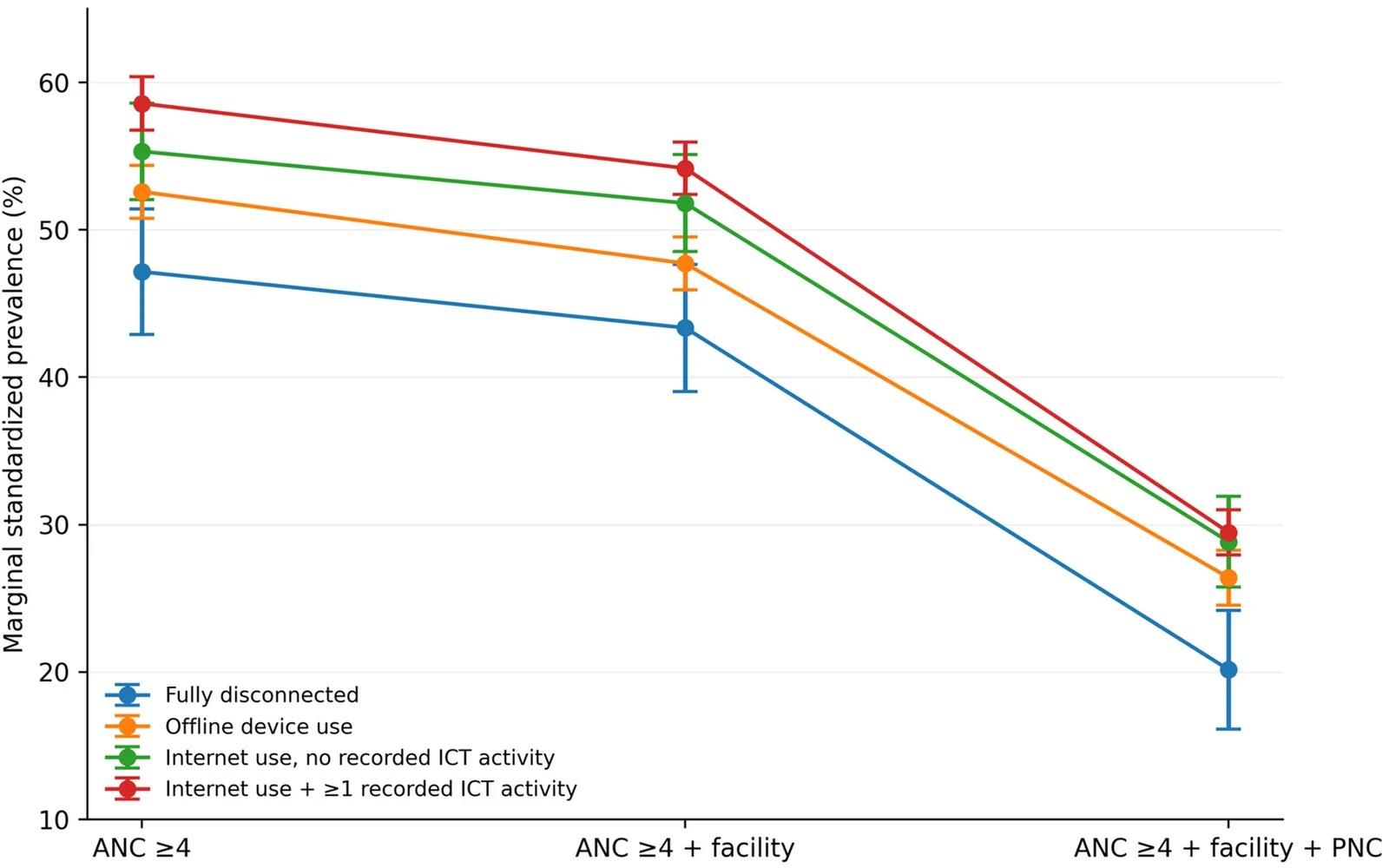
Adjusted cumulative maternal-care cascade by recent digital-access category. Points show marginal standardized prevalences at successive stages of the maternal-care continuum—ANC ≥4, ANC ≥4 plus facility delivery, and ANC ≥4 plus facility delivery plus maternal PNC; vertical error bars show 95% confidence intervals.

Conditional estimates further showed that facility delivery was common once ANC ≥4 had been achieved: 89.5% among fully disconnected women, 88.1% with offline device use, 92.2% among Internet users without recorded ICT activity, and 94.4% in the reference category. Among women who achieved both ANC ≥4 and facility delivery, subsequent PNC prevalence was 39.8%, 50.4%, 50.1%, and 58.7%, respectively. The largest separation between digital-access groups was therefore already evident at the ANC stage, with further attrition occurring at PNC.

### ANC ≥8 and exploratory digital-health indicators

ANC ≥8 was attained by 19 of 876 fully disconnected women, 216 of 4,070 women with offline device use, 74 of 1,123 Internet users without recorded ICT activity, and 839 of 4,654 women in the reference category. Corresponding survey-weighted prevalences were 2.95% (95% CI 1.54–4.35), 6.87% (5.93–7.80), 7.96% (6.02–9.90), and 20.13% (18.66–21.59). In adjusted analysis, the aPR for ANC ≥8 was 0.43 (95% CI 0.27–0.71) among fully disconnected women, 0.74 (0.62–0.88) with offline device use, and 0.70 (0.54–0.91) among Internet users without recorded ICT activity. Because only 19 fully disconnected women attained ANC ≥8, this secondary endpoint was interpreted cautiously; adjusted results for ANC ≥8 and the other secondary maternal-care outcomes are provided in Supplementary Table S2.

Health-related Internet behaviors were uncommon: 428 women reported online health-information seeking (survey-weighted 4.3%) and 225 reported an online healthcare appointment (2.0%). These variables were retained as descriptive exploratory behaviors rather than interpreted as mediators or mechanisms of the primary associations.

### Temporal data audit and robustness analyses

Audit of maternity-history dates confirmed substantial limitations in assigning a precise interval between the index birth and interview. Of 10,723 women in the primary cohort, 10,517 had a linked maternity-history record. Among these, 1,735 had an invalid or unavailable birth month; among records with a usable month, 370 births appeared more than 36 months before interview and nine fell after the interview month. Consequently, 2,320 women (21.6%) could not be assigned reliably to a precise 0–36-month interval using the released month/year fields. This proportion differed across digital-access categories—28.5% among fully disconnected women, 24.4% with offline device use, 22.7% among Internet users without recorded ICT activity, and 17.6% in the reference group—indicating that simple restriction to complete month-level dates could introduce differential selection.

The ANC ≥4 association was stable across the principal robustness analyses (Table 5), and the primary estimates were essentially unchanged when socioeconomic position was alternatively classified using survey-weighted rank-based consumption quintiles (Supplementary Table S4). With child-birth-year fixed effects, the aPR for fully disconnected women was 0.75 (95% CI 0.66–0.85); in the stricter internally consistent birth-date subset it was 0.72 (0.63–0.83). Additional adjustment for decision-making and household media availability yielded 0.77 (0.68–0.86), while the combined social-context plus birth-year model yielded 0.76 (0.67–0.86). Inverse-probability weighting for complete-case participation yielded 0.75 (0.67–0.85). When province fixed effects were replaced by 158 HIES sampling-stratum fixed effects, the ANC association attenuated but remained evident (0.83, 0.74–0.93).

**Table 5:** Sensitivity analyses for fully disconnected women compared with internet use plus ≥1 recorded ICT activity.

Sensitivity specification	n (ANC/facility/PNC)	ANC ≥4 aPR (95% CI)	Facility-delivery aPR (95% CI)	PNC aPR (95% CI)
Primary model	10,496/10,496/10,434	0.75 (0.67–0.85)	0.94 (0.89–1.00)	0.87 (0.76–0.99)
Categorical age/parity specification	10,496/10,496/10,434	0.75 (0.67–0.85)	0.94 (0.89–1.00)	0.87 (0.76–0.99)
+ decision-making/media context	10,492/10,492/10,430	0.77 (0.68–0.86)	0.95 (0.90–1.00)	0.86 (0.76–0.98)
Child-birth-year fixed effects	9,868/9,868/9,811	0.75 (0.66–0.85)	0.95 (0.90–1.01)	0.87 (0.76–0.99)
Strict internally consistent birth-date subset	8,369/8,369/8,336	0.72 (0.63–0.83)	0.95 (0.89–1.01)	0.90 (0.78–1.03)
Combined social-context + birth-year model	9,864/9,864/9,807	0.76 (0.67–0.86)	0.96 (0.91–1.01)	0.86 (0.76–0.99)
Inverse-probability complete-case weighting	10,496/10,496/10,434	0.75 (0.67–0.85)	0.94 (0.89–1.00)	0.87 (0.77–0.99)
Missing-category analysis	10,723/10,723/10,660	0.77 (0.68–0.86)	0.94 (0.89–0.99)	0.88 (0.77–0.99)
158 sampling-stratum fixed effects	10,496/10,496/10,434	0.83 (0.74–0.93)	0.98 (0.92–1.03)	0.94 (0.83–1.07)

Values are aPRs (95% CIs) for the fully disconnected category relative to Internet use with ≥1 recorded ICT activity. The combined model included the primary-model covariates, medical-treatment decision-making, employment decision-making, household radio/television availability, and child-birth-year fixed effects. Stratum fixed effects provide finer geographic adjustment but are not direct measures of facility readiness, provider density, or policy exposure.

The facility-delivery association was more specification-sensitive: its confidence interval included 1.0 after social-context adjustment, birth-year adjustment, strict-date restriction, the combined social-context plus birth-year model, and stratum fixed effects. The PNC association remained below 1.0 in several temporal and social-context models but attenuated to 0.94 (95% CI 0.83–1.07) with stratum fixed effects and 0.90 (0.78–1.03) in the strict-date subset. Thus, the robustness analyses distinguished a comparatively stable ANC association from weaker evidence for facility delivery and less stable evidence for PNC.

### Geographic heterogeneity

Secondary interaction analyses indicated geographic heterogeneity in the association between digital-access category and ANC ≥4 (digital access  ×  province *P* < 0.001), facility delivery (*P* = 0.006), and PNC (*P* = 0.007). Rural/urban heterogeneity was also observed for ANC ≥4 (*P* = 0.002) and facility delivery (*P* = 0.015), but not PNC (*P* = 0.256). Province-specific adjusted estimates are reported in Supplementary Table S5 so that the interaction tests can be evaluated against the underlying estimates. Some provincial cells, particularly among fully disconnected women in Balochistan and for ANC ≥8, were sparse and were therefore interpreted cautiously.

## Discussion

### Principal findings

In this population-based analysis of 10,723 women from Pakistan's four provinces who reported a live birth during the preceding three years, recent digital access was strongly socially patterned and was associated with maternal healthcare utilization, although the magnitude and robustness of the association differed substantially across stages of care. The clearest finding involved antenatal care (ANC). Compared with women who used the Internet and reported at least one of the specified ICT activities, fully disconnected women had approximately 25% lower adjusted prevalence of receiving at least four ANC contacts, corresponding to an adjusted absolute difference of 11.4 percentage points. This association remained evident after alternative modeling of maternal age and lifetime live births, additional adjustment for women's decision-making and household media resources, child-birth-year adjustment, alternative approaches to missing data, restriction to women with internally consistent birth dates, and finer geographic adjustment. In contrast, the association with facility delivery was small and sensitive to model specification, while the association with maternal postnatal care (PNC) was weaker and attenuated under the strictest temporal and geographic analyses.

These findings should be interpreted within the broader socioeconomic patterning of maternal healthcare in Pakistan. Previous national studies have consistently identified household economic position, maternal education, residence, parity, women's autonomy, media exposure, and geography as important correlates of ANC, delivery care, PNC, and completion of the maternal-care continuum (5–8, 43). Systematic reviews from LMICs and South Asia report similar inequalities according to socioeconomic position, education, and place of residence (3, 4). In the present analysis, the marked attenuation of crude associations after adjustment indicates that a substantial proportion of the observed digital gradient overlaps with these established social and geographic inequalities. The remaining estimates therefore represent conditional associations after adjustment for measured covariates and should not be interpreted as isolated or causal effects of digital connectivity.

### Antenatal care: the most robust association

The concentration of the strongest and most reproducible association at the ANC stage is plausible because antenatal care requires repeated, planned contact over several months. Digital access could potentially support pregnancy-related information seeking, communication with family or healthcare providers, reminders, appointment planning, and navigation of available services. These mechanisms are consistent with the wider digital-health literature, but they were not measured directly in HIES and therefore remain plausible explanations rather than demonstrated mediators (9–11, 18, 19).

Evidence from mHealth interventions provides external support for the plausibility of these pathways but should not be equated with the present observational association. Rahman et al. reported that one-way and two-way mobile-health communication strategies improved attendance at four or more ANC contacts in randomized and cluster-randomized studies from LMICs (26). Mishra et al. similarly found that mHealth interventions can improve antenatal monitoring and care attendance (27), while a broader systematic review by Knop et al. identified ANC attendance among the maternal and child outcomes with comparatively consistent evidence of benefit (28). These studies evaluated health-specific interventions, whereas HIES measures general recent patterns of device and Internet use. Women in the present study were not assigned to a digital intervention, and their current digital circumstances may primarily reflect broader social and material resources.

Observational evidence on Internet use also supports cautious interpretation. Suziedelyte found that online health-information seeking was associated with greater use of professional healthcare, suggesting that Internet information may complement rather than necessarily substitute for formal care (16). In India, Krishnatri and Vellakkal used a telecommunications-market disruption to study affordable Internet expansion and reported increased use of ANC, institutional delivery, PNC, and modern contraception (17). Pregnancy-specific research likewise indicates that women frequently use Internet sources alongside professional advice, while also experiencing uncertainty and information burden (18, 19). Agreement in direction across these different studies increases the plausibility of a relationship between digital circumstances and healthcare use, but differences in design, setting, exposure definition, and causal identification preclude direct causal inference from the HIES estimates.

The ANC ≥8 analysis provides additional context. Eight or more contacts were uncommon overall and particularly rare among fully disconnected women, among whom only 19 attained this threshold. The direction of association was consistent with the primary ANC ≥4 finding, but the small event count reduced precision. Using ANC ≥4 as the primary utilization outcome while presenting ANC ≥8 as a secondary analysis therefore provides a more stable principal estimate while acknowledging the contemporary WHO eight-contact framework (2).

### Facility delivery: a small and context-sensitive association

The facility-delivery finding warrants more cautious interpretation. In the primary model, complete disconnection was associated with only a small relative difference in facility delivery, and the confidence interval was close to the null. The estimate attenuated further following additional social-context adjustment, child-birth-year adjustment, restriction to women with internally consistent birth dates, and particularly after replacement of province fixed effects with 158 HIES sampling-stratum fixed effects. This pattern suggests that the association with delivery location is more sensitive to local context than the association with ANC.

There are plausible reasons for this difference. Facility delivery was already common in the study population, leaving less scope for large between-group differences. Intervention evidence also suggests that digital approaches may produce less consistent changes in facility delivery than in ANC attendance, particularly where baseline facility use is already high (26, 28).

More importantly, delivery location depends strongly on factors that digital access alone cannot address. Mixed-methods research from rural Pakistan has identified transportation, cost, family decision-making, knowledge of available services, and perceived quality as important determinants of continuity of maternity care (29). Health-financing and policy environments may also influence utilization. Evidence from Punjab has linked public insurance expansion with increased hospital delivery and skilled attendance in program areas (30), while national survey analyses have reported higher maternal-health service utilization among women with insurance coverage after socioeconomic adjustment (31). These findings reinforce the possibility that health-system and policy factors may confound associations between digital circumstances and facility use.

Facility delivery should also be distinguished clearly from quality of care. Physical attendance at a healthcare facility does not guarantee technically competent, safe, respectful, or person-centered maternity care (32, 33). HIES identifies the place of delivery but does not measure emergency obstetric readiness, clinical quality, respectful care, treatment appropriateness, or maternal and neonatal outcomes. The present finding should therefore be interpreted strictly as an association with facility utilization, not with quality of maternity care.

### Postnatal care and the sequential maternal-care cascade

Maternal PNC showed a different pattern from ANC. Complete disconnection was associated with lower PNC in the primary model, whereas women with offline device use and Internet users without a recorded ICT activity were similar to the reference group. The estimate for fully disconnected women also moved toward the null under fine-geographic adjustment and restriction to women with internally consistent birth dates. The PNC finding therefore appears less robust than the ANC association.

This is consistent with the complex determinants of postpartum care. Pakistani research indicates that maternal and newborn PNC are shaped by education, household resources, mode and place of delivery, institutional contact, community characteristics, and other multilevel factors (34). A Pakistan–Bangladesh comparison likewise demonstrated substantial socioeconomic inequalities in maternal PNC utilization (35). Personal access to a phone could plausibly facilitate communication, reminders, or follow-up during the short postpartum period, but HIES does not measure provider messaging, reminder receipt, emergency communication, or the device used to access the Internet. Any such explanation therefore remains hypothesis-generating.

The sequential care-cascade analysis adds important resolution. Differences between digital-access categories were already evident at ANC ≥4, while facility delivery was common among women who had reached that ANC threshold. Considerable attrition was then observed between delivery and maternal PNC. Earlier Pakistani studies have similarly documented loss across successive stages of the maternal-care continuum and strong socioeconomic patterning of completion (6, 7, 34, 35). These findings support a stage-specific interpretation: recent digital access is not associated with an equal difference at every point in the maternal-care pathway, and a single all-or-none continuum measure would obscure where separation occurs.

The scope of the PNC outcome should also remain explicit. HIES asks whether the woman herself received a postnatal check-up within six weeks. The released maternity module does not provide a separate comparable newborn-PNC variable. The present findings therefore concern maternal PNC only and should not be generalized to newborn postnatal-care utilization.

### Socioeconomic patterning and effect modification

A particularly important finding was the steep socioeconomic patterning of digital access itself. Internet use with at least one recorded ICT activity was uncommon among women in the poorest consumption quintile but predominated among women in the richest quintile, while complete disconnection followed the opposite pattern. This is consistent with previous Pakistani evidence showing marked gender, educational, and rural–urban inequalities in mobile-phone ownership (14) and with multicountry evidence showing that women who are poorer or live in rural areas are less likely to own mobile phones (15).

The digital-access  ×  consumption-quintile interaction for ANC should be interpreted as heterogeneity in an observational association rather than as evidence of differential treatment effects. The adjusted ANC difference between fully disconnected women and the reference digital-access category was larger among women in the poorest quintile than among those in the richest quintile. However, neither socioeconomic position nor digital access was randomized. Digital-health-equity frameworks emphasize that digital participation depends on interrelated factors including education, affordability, language, autonomy, privacy, social support, and service design (9, 10, 36, 37). The observed interaction may therefore reflect different combinations of these constraints and resources across socioeconomic groups rather than a greater causal benefit of connectivity among poorer women.

Digital access should consequently not be viewed as an alternative explanation to socioeconomic inequality. Rather, recent digital disconnection appears to identify women who are also disproportionately exposed to established economic and geographic disadvantages. Digital-health literacy itself is socially patterned by education, income, age, and social support (38), and research among digitally marginalized populations demonstrates that device ownership does not necessarily translate into meaningful or independent access to digital health services (39, 40).

### Interpreting the four digital-access categories

The four HIES categories should be interpreted as nominal patterns of recent access and use, not as an ordered scale of digital competence or literacy. Women classified as Internet users without one of the 11 specified ICT activities may engage in messaging, social media, entertainment, health information seeking, or other online activities not represented by those items. Conversely, reporting one or more specified activities does not demonstrate advanced digital ability. This distinction is consistent with digital-equity frameworks that separate material access, connectivity, skills, effective use, and the capacity to obtain meaningful benefit from digital systems (9, 10, 36–40).

The distinction between use and ownership is also important. Approximately half of Internet-using women in the study did not personally own a phone. HIES does not identify the exact device or ownership arrangement used during each Internet session, so these women cannot be classified definitively as shared-phone users. Nevertheless, the finding demonstrates that personal ownership and Internet participation are not interchangeable. Earlier Pakistani and multicountry evidence similarly shows that ownership measures do not capture the full complexity of women's access to mobile and digital resources (14, 15).

### Potential benefits and harms of digital connectivity

Digital connectivity should not be framed as uniformly beneficial. Internet access can broaden opportunities for obtaining information and communicating with formal healthcare services, but the quality and volume of information encountered online vary substantially. Jiang and Beaudoin showed that Internet use may support health literacy while perceived information overload can impede it (19). Pregnancy-specific studies have likewise reported both reassurance and anxiety associated with online information seeking (18), and misinformation has been documented in pregnancy-related social-media content (20).

The present findings therefore should not be interpreted as evidence that greater Internet use necessarily improves maternal healthcare. Digital access may facilitate useful communication or information seeking, but it may also expose users to inaccurate information, create information overload, reinforce misconceptions, or in some situations substitute for professional advice. Conversely, evidence that online health-information seeking may complement professional healthcare illustrates that these pathways need not be mutually exclusive (16). Which pathway predominates is likely to depend on information quality, digital health literacy, trust, privacy, and integration with formal services. HIES does not measure the quality, source, credibility, or clinical relevance of online information, and these mechanisms cannot therefore be distinguished in the present analysis.

### Geographic context, health-system factors, and residual confounding

Fine-geographic analysis provides an important qualification to the primary findings. Replacing province with 158 sampling-stratum fixed effects attenuated the ANC association but did not remove it, while estimates for facility delivery and PNC moved considerably closer to the null. This pattern suggests that local contextual factors may be particularly influential for delivery and postpartum care.

Sampling-stratum fixed effects should nevertheless not be interpreted as direct measures of healthcare-system conditions. They absorb finer geographic variation than province indicators but do not measure facility readiness, provider density, travel time, emergency obstetric capacity, transport availability, healthcare quality, or local program exposure. Because the released geographic structure does not provide a uniform district identifier suitable for consistent linkage across all four provinces, external district-level policy linkage could itself introduce geographic misclassification. Residual confounding by local healthcare supply, financing, accessibility, and quality therefore remains plausible.

The attenuation of facility-delivery and PNC associations after finer geographic adjustment is itself informative. It suggests that local healthcare and geographic circumstances account for more of the observed variation in these outcomes than they do for ANC, reinforcing the need to interpret each maternal-care outcome separately.

### Temporality, reverse causality, and missing-data considerations

The principal limitation for causal interpretation is temporal ordering. The ICT variables describe digital access during the three months preceding interview, whereas maternal-care outcomes may refer to a birth occurring up to three years earlier. Current digital status therefore cannot be assumed to represent digital circumstances during pregnancy. Postpartum changes in employment, household resources, residence, household composition, phone ownership, or Internet use may also have occurred after the birth of index.

The birth-date audit further showed that exact month-level recency could not be reconstructed reliably for a substantial minority of women and that date completeness differed across digital-access categories. Restricting the main analysis to women with complete dates could therefore have improved temporal precision while introducing differential selection. Child-birth-year fixed effects and the internally consistent birth-date subset were consequently used as complementary sensitivity analyses rather than replacing the primary cohort. The ANC association remained evident under both approaches, whereas facility-delivery and PNC estimates were less stable.

The direction of bias from this temporal mismatch cannot be specified with confidence. Misclassification of pregnancy-period digital access by current digital status may attenuate associations under some conditions, but nondifferential misclassification does not invariably bias estimates toward the null, particularly for multilevel or imperfectly classified exposures (41). If postpartum changes in digital circumstances are related to earlier maternal-care use, bias could also operate in the opposite direction. Associational terminology is therefore essential.

Missing covariate data was limited, and inverse-probability-of-complete-case weighting produced estimates similar to the primary analysis. Nevertheless, inverse-probability weighting depends on correct modeling of observation probabilities and cannot remove bias arising from unmeasured causes of missingness (42). The missing data analyses should therefore be interpreted as robustness checks rather than evidence that missingness is inconsequential.

### Implications for policy and future research

The policy implication is not that smartphones or Internet access should be treated as stand-alone maternal-health interventions. A more defensible interpretation is that digital transformation should be implemented in ways that do not create an additional barrier for women who are already socioeconomically or geographically disadvantaged.

In Pakistan, equity-oriented digital maternal-health strategies could include low-bandwidth SMS and voice options alongside smartphone applications; Urdu and relevant regional-language content; assisted, telephone, and face-to-face routes for registration, appointments, referral, and information; and systems that do not require personal smartphone ownership or continuous Internet connectivity. Voice-based approaches may be particularly relevant in settings where independent digital access is limited; research from rural Sindh has already examined women's access to and willingness to receive voice-message interventions to support ANC attendance (22). Digital communication should complement rather than replace functioning clinical, referral, and community-based services.

Future research should improve both temporal measurement and causal identification. Prospective pregnancy cohorts could repeatedly measure personal device ownership, independent or shared access, Internet use, digital health literacy, online health-information exposure, and maternal-service contacts throughout pregnancy and the postpartum period. Quasi-experimental evaluations of broadband or mobile-network expansion may provide stronger causal leverage, as illustrated by the Indian telecommunications study (17). Linking individual data to facility readiness, provider density, transport accessibility, and health-financing programs would also help distinguish digital factors from local healthcare supply. Future studies should additionally examine whether digital services improve not only contact with healthcare but also quality, respectful care, continuity, and maternal and neonatal outcomes.

### Strengths and limitations

This study has several strengths. It uses a large and recent four-province population-based survey and preserves the complex sampling design throughout estimation. HIES provides detailed individual ICT measures together with maternity-care outcomes, household consumption, education, employment, residence, decision-making, and household media resources. The analysis distinguishes the primary model from targeted sensitivity analyses, models maternal age nonlinearly and lifetime live births continuously, reports both relative and standardized absolute measures, formally evaluates socioeconomic effect modification, and examines temporal, missing-data, social-context, and geographic robustness. The sequential care-cascade analysis also provides greater clinical and policy resolution than an all-or-none continuum indicator alone.

Several limitations remain. First, the cross-sectional design and temporal mismatch between current ICT measurement and earlier pregnancy care preclude causal inference and permit reverse temporal ordering. Second, birth-month information was incomplete or internally inconsistent for a substantial minority of women and differed in completeness across digital-access categories. Third, maternal-care utilization was self-reported and may be affected by recall or reporting error. Fourth, the constructed digital-access categories are based on available HIES indicators and are not validated measures of digital literacy, competence, independent access, or health-information exposure.

Fifth, residual confounding remains possible despite extensive adjustment. HIES does not directly measure maternity-facility readiness, provider density, travel time, transport availability, service quality, or consistently linkable local health-financing and service-delivery programs. Sixth, women's decision-making and household media variables were measured at interview and may themselves have changed since pregnancy; their inclusion therefore provides a sensitivity analysis rather than definitive confounding control. Seventh, the primary analyses used complete cases. Although missingness was limited and alternative missing-data analyses produced similar estimates, bias due to unmeasured determinants of missingness cannot be excluded (42). Eighth, facility delivery measures utilization rather than quality or respectful care (32, 33). Ninth, the PNC outcome applies only to the mother and does not provide a corresponding newborn-PNC endpoint. Finally, ANC ≥4 represents utilization rather than the full content or quality of antenatal care; ANC ≥8 was assessed secondarily, but sparse events among fully disconnected women reduced precision.

Taken together, these limitations warrant particular caution when interpreting facility delivery and PNC. The ANC association, however, remained comparatively stable across multiple alternative specifications and therefore represents the most consistent finding of the study.

## Conclusion and future directions

Among Pakistani women who had given birth within the preceding three years, complete digital disconnection was consistently associated with lower antenatal-care utilization. This association remained evident across alternative model specifications, including additional adjustment for social-context variables, adjustment for year of childbirth, alternative approaches to missing data, restriction to women with internally consistent birth dates, and finer geographic adjustment. By contrast, associations with facility delivery and maternal PNC were smaller and less consistent across analyses, indicating that the relationship between recent digital access and maternal healthcare varied across stages of care.

Digital access was also strongly socioeconomically patterned, and its association with ANC differed across household consumption groups. Together, these findings suggest that complete digital disconnection may identify women experiencing overlapping socioeconomic, geographic, and healthcare disadvantages. However, they do not establish that improving digital access would itself increase maternal-care utilization, particularly because digital circumstances were measured close to the survey interview and may have changed after the index pregnancy.

As maternal-health systems become increasingly digital, these findings support the importance of designing services that remain accessible to women with limited connectivity, no personal device, or fewer socioeconomic resources. Digital approaches should complement rather than replace accessible face-to-face and community-based care. Future prospective studies should measure digital circumstances during pregnancy, distinguish personal from shared access, and incorporate digital literacy and local health-system characteristics. Such studies could help determine whether digital pathways contribute independently to continuity of maternal care and which forms of digital support are most equitable.

## Data Availability

The data analyzed in this study is subject to the following licenses/restrictions: The HIES 2024–25 microdata analyzed in this study were obtained from the Pakistan Bureau of Statistics and are subject to PBS data-access procedures and terms of use. The authors do not have authority to redistribute the raw microdata. Analytical code and derived non-identifiable outputs are available from the corresponding authors on reasonable requests, subject to applicable PBS conditions. Requests to access these datasets should be directed to JA, muhammedjunaid.asghar@neu.edu.tr.
