# Supplementary material for: Digital access and maternal healthcare inequalities in Pakistan, 2024–2025: a population-based cross-sectional study
Source: Front Reprod Health. 2026 Oct 9;8:1976275. doi: 10.3389/frph.2026.1976275 (PMC13692637; doi:10.3389/frph.2026.1976275)
Supplement: Supplementary file 1 [file Datasheet1.pdf]

Supplementary Material

Supplementary Methods

Digital-position definition. The HIES ICT module uses a three-month reference period. The four mutually exclusive categories were: (1) fully disconnected; (2) offline device access; (3) Internet use without recorded ICT activity; and (4) Internet use with ≥1 recorded ICT activity. The 11 recorded activities are selected ICT behaviors and were not interpreted as a validated digital-literacy scale.

Survey design and inference. All analyses used the roster sampling weight without rescaling and treated the maternal cohort as a survey subpopulation/domain. Variance estimation preserved the full four-province structure of 2,153 PSUs in 158 strata (design df=1,995), with nine singleton strata handled using the centered approach. Primary aPRs and interaction tests were estimated using modified Poisson regression with design-based robust covariance. Absolute adjusted prevalences reported in the main manuscript were obtained separately from survey-weighted logistic models with marginal standardization because logistic predictions remain within the probability scale.

Household consumption reconstruction. Monthly per-capita consumption was reconstructed from the corrected HIES Section 6 microdata by harmonizing the component-specific recall periods to monthly equivalents and dividing by household size. During the data audit, inclusion of the durable-purchase aggregate (code 6000) produced substantially poorer agreement with PBS published welfare aggregates; the primary reconstruction therefore excluded code 6000 and applied the official HIES 2024–25 quintile thresholds. A survey-weighted rank-quintile sensitivity analysis was prespecified to test dependence on this reconstruction and produced essentially unchanged primary aPR estimates (Table S4).

Temporal sensitivity. A planned restriction to births within ≤12 or ≤24 months was not implemented because the released maternity-history month/year fields contained substantial missingness and internal inconsistencies. Retaining the documented three-year maternity-module eligibility window was judged preferable to constructing potentially misclassified recency samples. This temporal mismatch is treated as a limitation in the main manuscript.

Table S1. Component-specific digital exposure sensitivity analyses

Exposure	Outcome	n	aPR (95% CI)	P value
No Internet use	ANC ≥4	10496	0.88 (0.84–0.92)	<0.001
No Internet use	Facility delivery	10496	0.97 (0.95–0.99)	0.011
No Internet use	PNC within 6 weeks	10434	0.98 (0.92–1.04)	0.520
No smartphone ownership	ANC ≥4	10496	0.94 (0.90–0.98)	0.008
No smartphone ownership	Facility delivery	10496	0.97 (0.95–0.99)	<0.001
No smartphone ownership	PNC within 6 weeks	10434	0.98 (0.92–1.04)	0.471
No personal phone ownership	ANC ≥4	10496	0.92 (0.89–0.96)	<0.001
No personal phone ownership	Facility delivery	10496	0.96 (0.94–0.98)	<0.001
No personal phone ownership	PNC within 6 weeks	10434	0.91 (0.86–0.96)	<0.001

All estimates are fully adjusted using the prespecified covariate set. aPR = adjusted prevalence ratio; PNC = postnatal care.

Table S2. Secondary maternal healthcare outcomes by digital-inclusion stage

Outcome	Stage	n	aPR (95% CI)	P value
Any ANC	Fully disconnected	10496	0.89 (0.85–0.94)	<0.001
Any ANC	Offline device access	10496	0.96 (0.94–0.98)	<0.001
Any ANC	Internet use, no recorded ICT activity	10496	0.97 (0.94–1.00)	0.039
Early ANC (months 1–3)	Fully disconnected	10411	0.87 (0.79–0.96)	0.007
Early ANC (months 1–3)	Offline device access	10411	0.93 (0.89–0.98)	0.005
Early ANC (months 1–3)	Internet use, no recorded ICT activity	10411	0.90 (0.84–0.97)	0.004
ANC ≥8	Fully disconnected	10496	0.43 (0.27–0.71)	<0.001
ANC ≥8	Offline device access	10496	0.74 (0.62–0.88)	<0.001
ANC ≥8	Internet use, no recorded ICT activity	10496	0.70 (0.54–0.91)	0.008
Skilled birth attendance	Fully disconnected	10496	0.97 (0.92–1.02)	0.234
Skilled birth attendance	Offline device access	10496	0.97 (0.95–1.00)	0.041
Skilled birth attendance	Internet use, no recorded ICT activity	10496	0.99 (0.96–1.02)	0.496
ANC ≥4 + facility + PNC	Fully disconnected	10434	0.64 (0.50–0.81)	<0.001
ANC ≥4 + facility + PNC	Offline device access	10434	0.89 (0.80–0.98)	0.017
ANC ≥4 + facility + PNC	Internet use, no recorded ICT activity	10434	0.99 (0.86–1.13)	0.833

Reference category: Internet use with ≥1 recorded ICT activity. ANC ≥8 is a secondary outcome aligned with current WHO contact recommendations; sparse cells should be interpreted cautiously.

Table S3. Prespecified and secondary interaction tests

Interaction	Outcome	n	Wald χ^2	df	P value
Digital stage × consumption quintile	ANC ≥4	10496	28.86	12	0.004
Digital stage × consumption quintile	Facility delivery	10496	15.48	12	0.216
Digital stage × consumption quintile	PNC within 6 weeks	10434	11.02	12	0.527
Digital stage × province	ANC ≥4	10496	163.26	9	<0.001
Digital stage × province	Facility delivery	10496	23.09	9	0.006
Digital stage × province	PNC within 6 weeks	10434	22.86	9	0.007
Digital stage × rural/urban residence	ANC ≥4	10496	14.42	3	0.002
Digital stage × rural/urban residence	Facility delivery	10496	10.50	3	0.015
Digital stage × rural/urban residence	PNC within 6 weeks	10434	4.05	3	0.256

The digital stage × consumption quintile interaction was the prespecified primary interaction. Province and rural/urban interactions were secondary exploratory analyses; no multiplicity adjustment was applied.

Table S4. Sensitivity analysis using survey-weighted rank-based consumption quintiles

Outcome	Stage	n	aPR (95% CI)	P value
ANC ≥4	Fully disconnected	10496	0.75 (0.67–0.85)	<0.001
ANC ≥4	Offline device access	10496	0.89 (0.85–0.94)	<0.001
ANC ≥4	Internet use, no recorded ICT activity	10496	0.95 (0.89–1.03)	0.201
Facility delivery	Fully disconnected	10496	0.94 (0.89–1.00)	0.039
Facility delivery	Offline device access	10496	0.97 (0.95–0.99)	0.018
Facility delivery	Internet use, no recorded ICT activity	10496	0.99 (0.95–1.02)	0.421
PNC within 6 weeks	Fully disconnected	10434	0.87 (0.76–0.99)	0.032
PNC within 6 weeks	Offline device access	10434	1.00 (0.94–1.07)	0.966
PNC within 6 weeks	Internet use, no recorded ICT activity	10434	1.01 (0.92–1.11)	0.773

Reference category: Internet use with ≥1 recorded ICT activity. This analysis tests whether primary estimates depend on the official-cutoff consumption classification.

Table S5. Province-specific adjusted digital-stage associations

Province	Outcome	Stage	n	aPR (95% CI)	P value
Khyber Pakhtunkhwa	ANC ≥4	Fully disconnected	2260	0.73 (0.58–0.92)	0.009
Khyber Pakhtunkhwa	ANC ≥4	Offline device access	2260	0.78 (0.68–0.89)	<0.001
Khyber Pakhtunkhwa	ANC ≥4	Internet use, no recorded ICT activity	2260	0.84 (0.66–1.07)	0.160
Khyber Pakhtunkhwa	Facility delivery	Fully disconnected	2260	0.89 (0.79–1.01)	0.069
Khyber Pakhtunkhwa	Facility delivery	Offline device access	2260	0.97 (0.92–1.03)	0.297
Khyber Pakhtunkhwa	Facility delivery	Internet use, no recorded ICT activity	2260	0.98 (0.91–1.06)	0.622
Khyber Pakhtunkhwa	PNC within 6 weeks	Fully disconnected	2260	0.58 (0.39–0.87)	0.009
Khyber Pakhtunkhwa	PNC within 6 weeks	Offline device access	2260	0.96 (0.79–1.16)	0.663
Khyber Pakhtunkhwa	PNC within 6 weeks	Internet use, no recorded ICT activity	2260	0.87 (0.64–1.18)	0.359
Punjab	ANC ≥4	Fully disconnected	4137	0.74 (0.61–0.89)	0.002
Punjab	ANC ≥4	Offline device access	4137	1.00 (0.95–1.06)	0.985
Punjab	ANC ≥4	Internet use, no recorded ICT activity	4137	1.04 (0.96–1.13)	0.293
Punjab	Facility delivery	Fully disconnected	4137	1.00 (0.92–1.08)	0.980
Punjab	Facility delivery	Offline device access	4137	0.99 (0.95–1.02)	0.434
Punjab	Facility delivery	Internet use, no recorded ICT activity	4137	1.02 (0.97–1.06)	0.517
Punjab	PNC within 6 weeks	Fully disconnected	4137	0.80 (0.63–1.02)	0.066
Punjab	PNC within 6 weeks	Offline device access	4137	0.97 (0.88–1.07)	0.499
Punjab	PNC within 6 weeks	Internet use, no recorded ICT activity	4137	1.01 (0.89–1.15)	0.903
Sindh	ANC ≥4	Fully disconnected	2821	0.93 (0.75–1.16)	0.546
Sindh	ANC ≥4	Offline device access	2821	0.69 (0.60–0.80)	<0.001
Sindh	ANC ≥4	Internet use, no recorded ICT activity	2821	0.78 (0.66–0.92)	0.003

Province	Outcome	Stage	n	aPR (95% CI)	P value
Sindh	Facility delivery	Fully disconnected	2821	0.99 (0.92–1.07)	0.824
Sindh	Facility delivery	Offline device access	2821	0.94 (0.90–0.99)	0.014
Sindh	Facility delivery	Internet use, no recorded ICT activity	2821	0.92 (0.86–0.98)	0.009
Sindh	PNC within 6 weeks	Fully disconnected	2821	1.09 (0.90–1.31)	0.394
Sindh	PNC within 6 weeks	Offline device access	2821	1.11 (0.99–1.25)	0.067
Sindh	PNC within 6 weeks	Internet use, no recorded ICT activity	2821	1.05 (0.89–1.23)	0.589
Balochistan	ANC ≥4	Fully disconnected	1278	0.15 (0.07–0.31)	<0.001
Balochistan	ANC ≥4	Offline device access	1278	0.73 (0.49–1.08)	0.113
Balochistan	ANC ≥4	Internet use, no recorded ICT activity	1278	0.85 (0.50–1.46)	0.553
Balochistan	Facility delivery	Fully disconnected	1278	0.77 (0.62–0.95)	0.016
Balochistan	Facility delivery	Offline device access	1278	0.84 (0.71–0.99)	0.035
Balochistan	Facility delivery	Internet use, no recorded ICT activity	1278	0.94 (0.78–1.13)	0.478
Balochistan	PNC within 6 weeks	Fully disconnected	1216	1.14 (0.84–1.54)	0.402
Balochistan	PNC within 6 weeks	Offline device access	1216	1.03 (0.79–1.34)	0.846
Balochistan	PNC within 6 weeks	Internet use, no recorded ICT activity	1216	1.38 (1.04–1.85)	0.028

Reference category: Internet use with ≥1 recorded ICT activity. Province-specific estimates are secondary and some cells are sparse, particularly in Balochistan.

Table S6. Unadjusted maternal healthcare prevalence by digital-inclusion stage

Outcome	Stage	n	Survey-weighted prevalence, %
ANC ≥4	Fully disconnected	876	31.9
ANC ≥4	Offline device access	4070	43.5
ANC ≥4	Internet use, no recorded ICT activity	1123	52.1
ANC ≥4	Internet use + ≥1 recorded ICT activity	4654	67.6
Facility delivery	Fully disconnected	876	71.4
Facility delivery	Offline device access	4070	77.0
Facility delivery	Internet use, no recorded ICT activity	1123	81.5
Facility delivery	Internet use + ≥1 recorded ICT activity	4654	90.2
PNC within 6 weeks	Fully disconnected	853	29.7
PNC within 6 weeks	Offline device access	4049	37.2
PNC within 6 weeks	Internet use, no recorded ICT activity	1120	39.3
PNC within 6 weeks	Internet use + ≥1 recorded ICT activity	4638	51.0

Values are unadjusted survey-weighted prevalences. Stage categories use the three-month HIES ICT reference period.

Table S7. Missingness and complete-case contribution to primary models

Variable / model	Overall n missing (%)	Stage 1	Stage 2	Stage 3	Stage 4
Lifetime number of live births	185 (1.73)	30	79	23	53
Education	42 (0.39)	1	11	3	27
PNC within 6 weeks	63 (0.59)	23	21	3	16
Digital stage	0	0	0	0	0
ANC ≥4 complete-case model	n=10,496	845	3,980	1,097	4,574
Facility-delivery complete-case model	n=10,496	845	3,980	1,097	4,574
PNC complete-case model	n=10,434	822	3,960	1,094	4,558

Stage 1 = fully disconnected; Stage 2 = offline device access; Stage 3 = Internet use without recorded ICT activity; Stage 4 = Internet use with ≥1 recorded ICT activity. Other principal covariates used in the primary models were complete. Complete-case counts reflect outcome-specific models after excluding missing covariate values.
